# Crystal structure of *N*-(7-di­bromo­methyl-5-methyl-1,8-naphthyridin-2-yl)benzamide–pyrrolidine-2,5-dione (1/1)

**DOI:** 10.1107/S2056989016019034

**Published:** 2017-01-01

**Authors:** Bang Zhong Wang, Jun Ping Zhou, Yong Zhou, Jian Song Luo, Jun Jie Yang, Shao M.ing Chi

**Affiliations:** a College of Chemistry and Chemical Engineering, Yunnan Normal University, Kunming 650500, People’s Republic of China

**Keywords:** crystal structure, 1,8-naphthyridine, hydrogen bonding, π–π inter­action

## Abstract

The title compound, C_17_H_13_Br_2_N_3_O·C_4_H_5_NO_2_, is a co-crystal of *N*-(7-di­bromo­methyl-5-methyl-1,8-naphthyridin-2-yl)benzamide and pyrrolidine-2,5-dione (succinimide). The benzamide mol­ecule exhibits pseudo-mirror symmetry, with an r.m.s. deviation of the non-H atoms of 0.09 Å (except for the two Br atoms). The angle between the least-squares planes of the two mol­ecules is 26.2 (2)°. In the crystal, the two mol­ecules are mutually linked by N—H⋯O and N—H⋯N hydrogen bonds. The packing is consolidated by C—H⋯(O,N) hydrogen bonds and π–π stacking inter­actions.

## Abstract 2

The title crystal is a co-crystal with the 1,8-naphthyridine derivative crystallizing with one mol­ecule of succinimide per formula unit. In the crystal, the two mol­ecules are mutually linked by N—H⋯O and N—H⋯N hydrogen bonds. The packing is consolidated by C—H⋯(O,N) hydrogen bonds and π–π stacking inter­actions.

## Chemical context   

1,8-Naphthyridine derivatives are important heterocyclic compounds that exhibit excellent biochemical and pharmacological properties. Moreover, these compounds benefit from conjugate π-electronic structures and are widely used as ligands in the synthesis of metal complexes (Tang *et al.*, 2015 ▸; Matveeva *et al.*, 2012 ▸, 2013 ▸), functional materials (Kuo *et al.*, 2011 ▸; Katz *et al.*, 2007 ▸; Hu & Chen, 2010 ▸) or as catalysts (Fuentes *et al.*, 2011 ▸; Yamazaki *et al.*, 2011 ▸). In a number of studies, the fluorescent properties of naphthyridines have been investigated (Yu *et al.*, 2013 ▸; Li *et al.*, 2012), in particular as selective fluorescent chemosensors for small biological mol­ecules through hydrogen bonding (Nakatani *et al.*, 2013 ▸; Liang *et al.*, 2012 ▸). 1,8-Naphthyridin–BF_2_ complexes are known to be fluorescent dyes with high chemical stability (Li *et al.*, 2014 ▸), high fluorescence quantum yields (Quan *et al.*, 2012 ▸), high extinction coefficients (Wu *et al.*, 2013 ▸) and sharp fluorescence peaks (Du *et al.*, 2014 ▸). Some anti­viral medications are also based on 1,8-naphthyridines (Elansary *et al.*, 2014 ▸). In this context we aimed to synthesize the title 1,8-naphthyridine derivative and report here on the crystal structure of the obtained co-crystal with pyrrolidine-2,5-dione (succinimide).
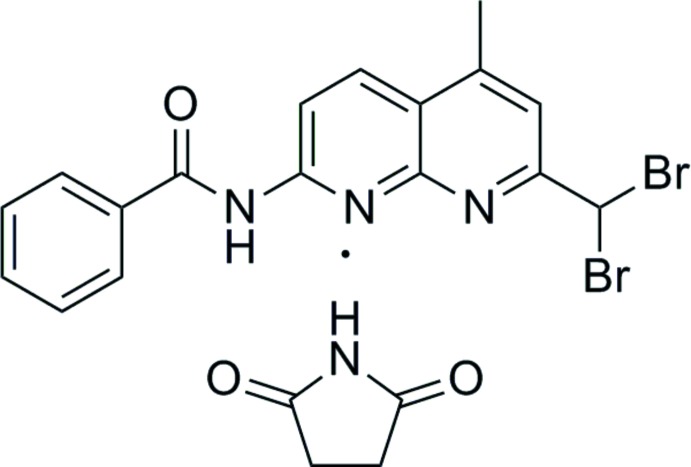


## Structural commentary   

**Figure 1:**
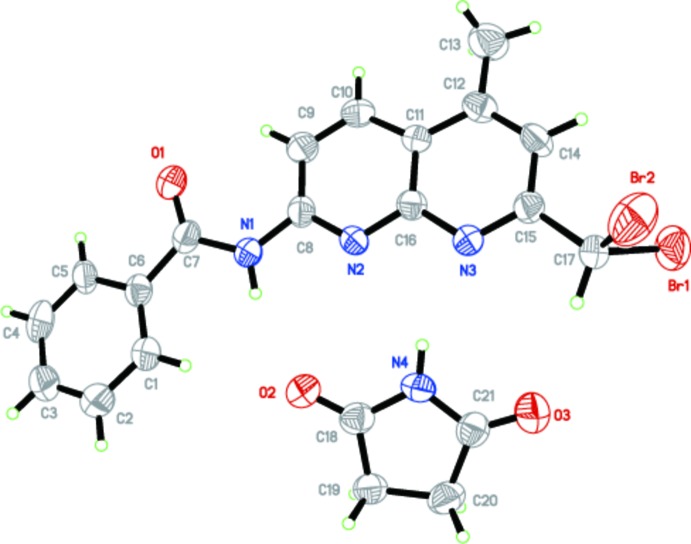
The mol­ecular components in the title co-crystal, showing the atom labelling. Displacement ellipsoids are drawn at the 50% probability level.

The mol­ecular structure of the title 1,8-naphthyridine deriv­ative is shown in Fig. 1 ▸. The *N*-(7-(di­bromo­meth­yl)-5-methyl-1,8-naphthyridin-2-yl)benzamide moiety (except the two Br atoms) is essentially planar (r.m.s deviation = 0.09 Å), with the maximum deviation from the mean plane being 0.315 (5) Å for atom O1. The naphthyridine ring system makes a dihedral angle of 2.2 (2)° with the benzene ring and is oriented at an angle of 26.2 (2)° relative to the succinimide. The conformation of the C=O and the N—H bonds of the amide segment are *anti* to one another, similar to that reported for benzamide moiety in *N*-{4-[(6-chloro­pyridin-3-yl)-meth­oxy]phen­yl}-2,6-di­fluoro­benzamide (Liang *et al.*, 2016 ▸).

## Supra­molecular features   

**Figure 2:**
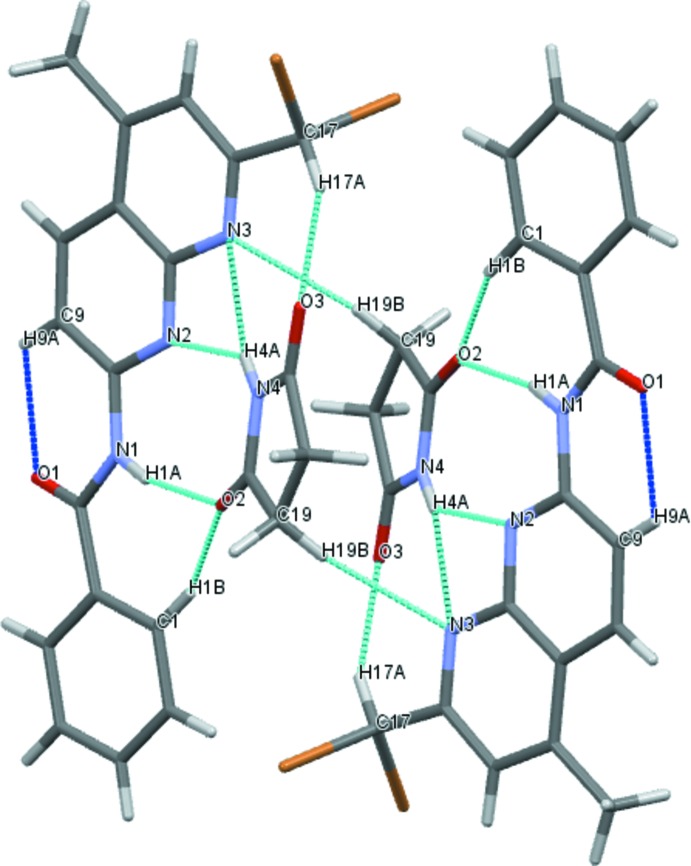
The different types of hydrogen bonds between the two mol­ecules and pairs of mol­ecules; intra­molecular hydrogen bonds are shown as blue dashed lines and inter­molecular hydrogen bonds are shown as turquoise dashed lines.

**Figure 3:**
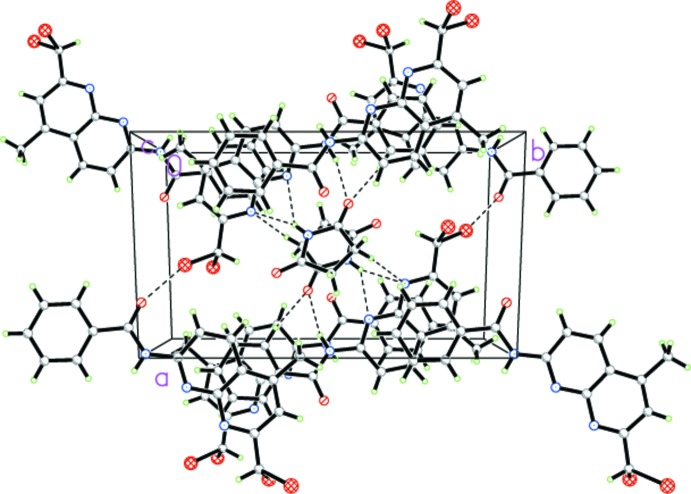
A view along the *c* axis, showing the crystal packing of the title compound.

The two mol­ecules are mutually linked into pairs by N—H⋯O and N—H⋯N hydrogen bonds with the (imide)N—H⋯N bond bifurcated (Table 1 ▸, Fig. 2 ▸). In the 1,8-naphthyridine derivative, an intra­molecular C—H⋯O hydrogen bond between a phenyl H atom and the carbonyl function is also present. Apart from the classical hydrogen-bonding inter­actions, the two mol­ecules are additionally linked by weaker C—H⋯O and C—H⋯N hydrogen bonds. These pairs are linked by weak C—Br⋯O inter­actions [3.094 (5) Å]. The supra­molecular aggregation is completed by π–π stacking inter­actions between two neighbouring succinimide mol­ecules with a centroid-to-centroid distance of *Cg*⋯*Cg* ^i^ = 3.854 (4) Å [inter­planar distance = 3.172 (3) Å; symmetry code: −*x* + 1, −*y* + 1, −*z* + 1], forming a three-dimensional supra­molecular network (Fig. 3 ▸).

## Database survey   

In the Cambridge Structural Database (Version 5.37; Groom *et al.*, 2016 ▸), the structural data for a very similar 1,8-naphthyridine deriv­ative have been deposited (CSD refcode LESBOC; Gou *et al.*, 2013 ▸). Instead of a benzamide, the latter is an acetamide where the dihedral angle between the naphthyridine moiety and the succinimide co-mol­ecule is 14.1°.

## Synthesis and crystallization   

*N*-(5,7-dimethyl-1,8-naphthyridin-2-yl)benzamide (Wu *et al.*, 2012 ▸) (0.277 g,1 mmol) and *N*-bromo­succinimide (0.356 g, 2 mmol) were added to an dry aceto­nitrile (30 ml) solution under nitro­gen atmosphere. The mixture was refluxed at room temperature in the presence of light with a 250 W infrared lamp for 4 h. Excess solvent was removed and the crude product was purified by column chromatography using di­chloro­methane/methanol (120:1) as the mobile phase to give a light-yellow powder (yield: 0.1 g; 19%). Crystals suitable for X-ray analysis were obtained by slow diffusion of a di­chloro­methane solution at ambient temperature. Several cycles of purification by chromatography were used to reduce the amount of succinimide.

## Refinement   

Crystal data, data collection and structure refinement details are summarized in Table 2 ▸. H atoms were constrained to an ideal geometry with C—H distances in the range 0.93–0.96 Å, *U*_iso_(H) = 1.5*U*_eq_(C) for methyl H atoms and *U*_iso_(H) = 1.2*U*_eq_(C) for all other H atoms, and with N—H = 0.86 Å, *U*_iso_(H) = 1.2*U*_eq_(N).

## Supplementary Material

Crystal structure: contains datablock(s) I. DOI: 10.1107/S2056989016019034/wm5334sup1.cif

Structure factors: contains datablock(s) I. DOI: 10.1107/S2056989016019034/wm5334Isup2.hkl

Supporting information file. DOI: 10.1107/S2056989016019034/wm5334Isup3.cml

CCDC reference: 1519551

Additional supporting information: crystallographic information; 3D view; checkCIF report

## Figures and Tables

**Table 1:** Hydrogen-bond geometry (Å, °)

*D*—H⋯*A*	*D*—H	H⋯*A*	*D*⋯*A*	*D*—H⋯*A*
N1—H1*A*⋯O2	0.86	2.22	3.060 (7)	164
N4—H4*A*⋯N2	0.86	2.48	3.195 (7)	141
N4—H4*A*⋯N3	0.86	2.27	3.098 (7)	162
C1—H1*B*⋯O2	0.93	2.43	3.299 (8)	156
C9—H9*A*⋯O1	0.93	2.30	2.870 (8)	119
C17—H17*A*⋯O3	0.98	2.60	3.504 (8)	154
C19—H19*B*⋯N3^i^	0.97	2.58	3.538 (8)	170

Symmetry code: (i) 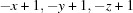.

**Table 2:** Experimental details

Crystal data	
Chemical formula	C_17_H_13_Br_2_N_3_O·C_4_H_5_NO_2_
*M*_r_	534.21
Crystal system, space group	Monoclinic, *P*2_1_/*c*
Temperature (K)	293
*a*, *b*, *c* (Å)	9.6931 (19), 15.699 (3), 14.614 (3)
β (°)	108.99 (3)
*V* (Å^3^)	2103.0 (7)
*Z*	4
Radiation type	Mo *K*α
μ (mm^−1^)	3.89
Crystal size (mm)	0.30 × 0.28 × 0.26

Data collection	
Diffractometer	Rigaku R-AXIS RAPID
Absorption correction	Multi-scan (*ABSCOR* ; Higashi, 1995 ▸)
*T*_min_, *T*_max_	0.389, 0.432
No. of measured, independent and observed [*I* > 2σ(*I*)] reflections	16558, 4129, 2010
*R*_int_	0.125
(sin θ/λ)_max_ (Å^−1^)	0.617

Refinement	
*R*[*F* ^2^ > 2σ(*F* ^2^)], *wR*(*F* ^2^), *S*	0.062, 0.148, 0.98
No. of reflections	4129
No. of parameters	271
H-atom treatment	H-atom parameters constrained
Δρ_max_, Δρ_min_ (e Å^−3^)	1.35, −0.43

Computer programs: *PROCESS-AUTO* (Rigaku, 1998 ▸), *CrystalStructure* (Rigaku/MSC, 2006 ▸), *SHELXS97* and *SHELXL97* (Sheldrick, 2008 ▸) and *DIAMOND* (Brandenburg, 1999 ▸).

## supplementary crystallographic information

### Crystal data

**Table:**

C_17_H_13_Br_2_N_3_O·C_4_H_5_NO_2_	*F*(000) = 1064
*M**_r_* = 534.21	*D*_x_ = 1.687 Mg m^−^^3^
Monoclinic, *P*2_1_/*c*	Mo *K*α radiation, λ = 0.71073 Å
*a* = 9.6931 (19) Å	Cell parameters from 4129 reflections
*b* = 15.699 (3) Å	θ = 3.1–26.0°
*c* = 14.614 (3) Å	µ = 3.89 mm^−^^1^
β = 108.99 (3)°	*T* = 293 K
*V* = 2103.0 (7) Å^3^	Block, white
*Z* = 4	0.30 × 0.28 × 0.26 mm

### Data collection

**Table:**

Rigaku R-AXIS RAPID diffractometer	4129 independent reflections
Radiation source: fine-focus sealed tube	2010 reflections with *I* > 2σ(*I*)
Graphite monochromator	*R*_int_ = 0.125
ω scans	θ_max_ = 26.0°, θ_min_ = 3.0°
Absorption correction: multi-scan (*ABSCOR* ; Higashi, 1995)	*h* = −11→11
*T*_min_ = 0.389, *T*_max_ = 0.432	*k* = −19→19
16558 measured reflections	*l* = −18→18

### Refinement

**Table:**

Refinement on *F*^2^	Primary atom site location: structure-invariant direct methods
Least-squares matrix: full	Secondary atom site location: difference Fourier map
*R*[*F*^2^ > 2σ(*F*^2^)] = 0.062	Hydrogen site location: inferred from neighbouring sites
*wR*(*F*^2^) = 0.148	H-atom parameters constrained
*S* = 0.98	*w* = 1/[σ^2^(*F*_o_^2^) + (0.0633*P*)^2^] where *P* = (*F*_o_^2^ + 2*F*_c_^2^)/3
4129 reflections	(Δ/σ)_max_ < 0.001
271 parameters	Δρ_max_ = 1.35 e Å^−^^3^
0 restraints	Δρ_min_ = −0.43 e Å^−^^3^

### Special details

**Table:**

Geometry. All esds (except the esd in the dihedral angle between two l.s. planes) are estimated using the full covariance matrix. The cell esds are taken into account individually in the estimation of esds in distances, angles and torsion angles; correlations between esds in cell parameters are only used when they are defined by crystal symmetry. An approximate (isotropic) treatment of cell esds is used for estimating esds involving l.s. planes.
Refinement. Refinement of F^2^ against ALL reflections. The weighted R-factor wR and goodness of fit S are based on F^2^, conventional R-factors R are based on F, with F set to zero for negative F^2^. The threshold expression of F^2^ > 2sigma(F^2^) is used only for calculating R-factors(gt) etc. and is not relevant to the choice of reflections for refinement. R-factors based on F^2^ are statistically about twice as large as those based on F, and R- factors based on ALL data will be even larger.

### Fractional atomic coordinates and isotropic or equivalent isotropic displacement parameters (Å^2^)

**Table:**

	*x*	*y*	*z*	*U*_iso_*/*U*_eq_	
Br1	0.58390 (8)	0.09771 (5)	0.60551 (6)	0.0662 (3)	
Br2	0.59853 (8)	0.16358 (6)	0.40343 (6)	0.0707 (3)	
O2	0.2878 (5)	0.5578 (3)	0.5158 (3)	0.0569 (13)	
N2	0.1482 (5)	0.3823 (3)	0.4170 (4)	0.0384 (13)	
N3	0.3233 (5)	0.2797 (3)	0.4629 (4)	0.0412 (13)	
C10	−0.0753 (6)	0.2634 (4)	0.3453 (5)	0.0472 (17)	
H10A	−0.1506	0.2244	0.3211	0.057*	
C16	0.1779 (6)	0.2982 (4)	0.4218 (4)	0.0372 (15)	
O3	0.6209 (5)	0.3572 (3)	0.6728 (4)	0.0670 (14)	
C11	0.0696 (6)	0.2340 (4)	0.3875 (4)	0.0386 (15)	
O1	−0.2418 (5)	0.5110 (3)	0.2818 (4)	0.0572 (13)	
C8	0.0100 (6)	0.4067 (4)	0.3782 (5)	0.0408 (15)	
N1	−0.0060 (5)	0.4946 (3)	0.3799 (4)	0.0431 (14)	
H1A	0.0691	0.5224	0.4148	0.052*	
C18	0.3969 (7)	0.5289 (4)	0.5745 (5)	0.0466 (17)	
C6	−0.1117 (7)	0.6382 (4)	0.3444 (4)	0.0403 (16)	
N4	0.4396 (5)	0.4441 (3)	0.5798 (4)	0.0440 (13)	
H4A	0.3908	0.4057	0.5406	0.053*	
C5	−0.2391 (7)	0.6855 (4)	0.3129 (5)	0.0472 (17)	
H5A	−0.3286	0.6584	0.2872	0.057*	
C12	0.1146 (6)	0.1482 (4)	0.3980 (5)	0.0438 (17)	
C3	−0.1020 (9)	0.8141 (5)	0.3572 (6)	0.065 (2)	
H3B	−0.1000	0.8733	0.3614	0.078*	
C7	−0.1274 (7)	0.5432 (4)	0.3325 (5)	0.0443 (17)	
C21	0.5664 (7)	0.4271 (5)	0.6533 (5)	0.0477 (17)	
C15	0.3601 (6)	0.1989 (4)	0.4687 (4)	0.0397 (15)	
C1	0.0213 (7)	0.6805 (4)	0.3830 (5)	0.0543 (19)	
H1B	0.1075	0.6494	0.4053	0.065*	
C4	−0.2326 (8)	0.7719 (5)	0.3199 (5)	0.063 (2)	
H4B	−0.3187	0.8031	0.2989	0.075*	
C19	0.5070 (6)	0.5760 (4)	0.6530 (5)	0.0468 (17)	
H19A	0.4625	0.6011	0.6971	0.056*	
H19B	0.5513	0.6209	0.6264	0.056*	
C14	0.2609 (7)	0.1317 (4)	0.4380 (5)	0.0463 (17)	
H14A	0.2943	0.0758	0.4447	0.056*	
C2	0.0253 (8)	0.7682 (5)	0.3881 (6)	0.071 (2)	
H2B	0.1143	0.7963	0.4125	0.085*	
C17	0.5246 (6)	0.1862 (4)	0.5102 (5)	0.0476 (18)	
H17A	0.5680	0.2398	0.5408	0.057*	
C9	−0.1054 (7)	0.3486 (4)	0.3399 (5)	0.0470 (17)	
H9A	−0.2005	0.3680	0.3116	0.056*	
C20	0.6209 (7)	0.5095 (4)	0.7050 (5)	0.0555 (19)	
H20A	0.7159	0.5240	0.7006	0.067*	
H20B	0.6286	0.5056	0.7727	0.067*	
C13	0.0061 (7)	0.0758 (5)	0.3690 (6)	0.070 (2)	
H13A	0.0573	0.0224	0.3811	0.105*	
H13B	−0.0487	0.0803	0.3014	0.105*	
H13C	−0.0592	0.0786	0.4062	0.105*	

### Atomic displacement parameters (Å^2^)

**Table:**

	*U*^11^	*U*^22^	*U*^33^	*U*^12^	*U*^13^	*U*^23^
Br1	0.0729 (5)	0.0613 (5)	0.0558 (5)	0.0189 (4)	0.0090 (4)	0.0111 (4)
Br2	0.0474 (4)	0.1006 (7)	0.0659 (6)	0.0050 (4)	0.0208 (4)	0.0021 (5)
O2	0.051 (3)	0.054 (3)	0.055 (3)	0.000 (2)	0.002 (3)	−0.002 (2)
N2	0.037 (3)	0.030 (3)	0.046 (3)	0.000 (2)	0.010 (3)	−0.001 (2)
N3	0.035 (3)	0.036 (3)	0.048 (4)	0.000 (3)	0.008 (3)	−0.001 (3)
C10	0.039 (4)	0.046 (4)	0.053 (5)	−0.010 (3)	0.009 (3)	−0.004 (3)
C16	0.035 (3)	0.038 (4)	0.040 (4)	0.001 (3)	0.014 (3)	−0.002 (3)
O3	0.068 (3)	0.054 (3)	0.069 (4)	0.009 (3)	0.009 (3)	0.006 (3)
C11	0.037 (3)	0.034 (4)	0.041 (4)	−0.001 (3)	0.008 (3)	−0.005 (3)
O1	0.042 (3)	0.053 (3)	0.063 (3)	0.009 (2)	−0.001 (3)	0.003 (3)
C8	0.039 (4)	0.039 (4)	0.043 (4)	0.006 (3)	0.011 (3)	0.004 (3)
N1	0.032 (3)	0.038 (3)	0.052 (4)	0.003 (2)	0.004 (3)	0.000 (3)
C18	0.041 (4)	0.050 (5)	0.051 (5)	−0.004 (4)	0.017 (4)	−0.003 (4)
C6	0.044 (4)	0.041 (4)	0.037 (4)	0.009 (3)	0.014 (3)	0.002 (3)
N4	0.047 (3)	0.038 (3)	0.045 (4)	−0.008 (3)	0.013 (3)	−0.006 (3)
C5	0.041 (4)	0.041 (4)	0.057 (5)	0.005 (3)	0.012 (3)	0.017 (3)
C12	0.044 (4)	0.042 (4)	0.050 (4)	−0.006 (3)	0.022 (3)	−0.007 (3)
C3	0.077 (5)	0.042 (5)	0.064 (5)	0.006 (4)	0.007 (4)	0.004 (4)
C7	0.037 (4)	0.049 (4)	0.044 (5)	0.005 (3)	0.008 (3)	0.008 (3)
C21	0.047 (4)	0.052 (5)	0.042 (4)	0.003 (4)	0.012 (4)	0.002 (4)
C15	0.042 (3)	0.041 (4)	0.035 (4)	0.004 (3)	0.009 (3)	−0.001 (3)
C1	0.046 (4)	0.044 (4)	0.063 (5)	0.006 (4)	0.004 (4)	0.007 (4)
C4	0.054 (5)	0.066 (6)	0.062 (6)	0.027 (4)	0.011 (4)	0.018 (4)
C19	0.042 (4)	0.046 (4)	0.051 (5)	−0.004 (3)	0.015 (3)	−0.005 (3)
C14	0.050 (4)	0.029 (4)	0.063 (5)	−0.002 (3)	0.023 (4)	−0.006 (3)
C2	0.062 (5)	0.050 (5)	0.078 (6)	0.001 (4)	−0.006 (4)	0.000 (4)
C17	0.043 (3)	0.036 (4)	0.052 (5)	0.006 (3)	0.001 (3)	0.000 (3)
C9	0.037 (3)	0.046 (5)	0.056 (5)	−0.002 (3)	0.012 (3)	−0.001 (4)
C20	0.046 (4)	0.062 (5)	0.051 (5)	−0.005 (4)	0.006 (4)	−0.007 (4)
C13	0.056 (5)	0.059 (5)	0.093 (7)	−0.009 (4)	0.023 (5)	−0.013 (4)

### Geometric parameters (Å, º)

**Table:**

Br1—C17	1.918 (6)	C5—C4	1.359 (9)
Br2—C17	1.950 (7)	C5—H5A	0.9300
O2—C18	1.213 (7)	C12—C14	1.372 (8)
N2—C8	1.330 (7)	C12—C13	1.513 (9)
N2—C16	1.348 (7)	C3—C2	1.373 (10)
N3—C15	1.312 (7)	C3—C4	1.375 (10)
N3—C16	1.372 (7)	C3—H3B	0.9300
C10—C9	1.366 (8)	C21—C20	1.505 (9)
C10—C11	1.415 (8)	C15—C14	1.399 (8)
C10—H10A	0.9300	C15—C17	1.524 (8)
C16—C11	1.424 (8)	C1—C2	1.379 (9)
O3—C21	1.211 (7)	C1—H1B	0.9300
C11—C12	1.408 (8)	C4—H4B	0.9300
O1—C7	1.225 (7)	C19—C20	1.529 (8)
C8—N1	1.390 (7)	C19—H19A	0.9700
C8—C9	1.410 (8)	C19—H19B	0.9700
N1—C7	1.383 (7)	C14—H14A	0.9300
N1—H1A	0.8600	C2—H2B	0.9300
C18—N4	1.389 (8)	C17—H17A	0.9800
C18—C19	1.485 (9)	C9—H9A	0.9300
C6—C5	1.385 (8)	C20—H20A	0.9700
C6—C1	1.396 (9)	C20—H20B	0.9700
C6—C7	1.505 (8)	C13—H13A	0.9600
N4—C21	1.370 (8)	C13—H13B	0.9600
N4—H4A	0.8600	C13—H13C	0.9600
			
C8—N2—C16	118.2 (5)	N3—C15—C17	112.3 (5)
C15—N3—C16	116.9 (5)	C14—C15—C17	123.4 (6)
C9—C10—C11	120.5 (6)	C2—C1—C6	120.1 (6)
C9—C10—H10A	119.8	C2—C1—H1B	119.9
C11—C10—H10A	119.8	C6—C1—H1B	119.9
N2—C16—N3	113.7 (5)	C5—C4—C3	121.7 (7)
N2—C16—C11	123.7 (5)	C5—C4—H4B	119.2
N3—C16—C11	122.6 (5)	C3—C4—H4B	119.2
C12—C11—C10	125.9 (6)	C18—C19—C20	105.4 (5)
C12—C11—C16	118.2 (5)	C18—C19—H19A	110.7
C10—C11—C16	115.9 (5)	C20—C19—H19A	110.7
N2—C8—N1	112.4 (5)	C18—C19—H19B	110.7
N2—C8—C9	122.8 (6)	C20—C19—H19B	110.7
N1—C8—C9	124.8 (5)	H19A—C19—H19B	108.8
C7—N1—C8	128.3 (5)	C12—C14—C15	120.1 (6)
C7—N1—H1A	115.8	C12—C14—H14A	119.9
C8—N1—H1A	115.8	C15—C14—H14A	119.9
O2—C18—N4	125.0 (6)	C3—C2—C1	120.1 (7)
O2—C18—C19	127.0 (6)	C3—C2—H2B	120.0
N4—C18—C19	107.9 (6)	C1—C2—H2B	120.0
C5—C6—C1	119.1 (6)	C15—C17—Br1	114.3 (5)
C5—C6—C7	116.6 (6)	C15—C17—Br2	108.3 (4)
C1—C6—C7	124.3 (6)	Br1—C17—Br2	110.3 (3)
C21—N4—C18	113.9 (6)	C15—C17—H17A	107.9
C21—N4—H4A	123.0	Br1—C17—H17A	107.9
C18—N4—H4A	123.0	Br2—C17—H17A	107.9
C4—C5—C6	119.7 (6)	C10—C9—C8	118.9 (6)
C4—C5—H5A	120.1	C10—C9—H9A	120.5
C6—C5—H5A	120.1	C8—C9—H9A	120.5
C14—C12—C11	117.9 (6)	C21—C20—C19	105.0 (5)
C14—C12—C13	120.4 (6)	C21—C20—H20A	110.8
C11—C12—C13	121.7 (6)	C19—C20—H20A	110.8
C2—C3—C4	119.3 (7)	C21—C20—H20B	110.8
C2—C3—H3B	120.3	C19—C20—H20B	110.8
C4—C3—H3B	120.3	H20A—C20—H20B	108.8
O1—C7—N1	122.0 (6)	C12—C13—H13A	109.5
O1—C7—C6	121.1 (6)	C12—C13—H13B	109.5
N1—C7—C6	116.8 (6)	H13A—C13—H13B	109.5
O3—C21—N4	124.9 (6)	C12—C13—H13C	109.5
O3—C21—C20	127.3 (7)	H13A—C13—H13C	109.5
N4—C21—C20	107.8 (6)	H13B—C13—H13C	109.5
N3—C15—C14	124.4 (6)		

### Hydrogen-bond geometry (Å, º)

**Table:**

*D*—H···*A*	*D*—H	H···*A*	*D*···*A*	*D*—H···*A*
N1—H1*A*···O2	0.86	2.22	3.060 (7)	164
N4—H4*A*···N2	0.86	2.48	3.195 (7)	141
N4—H4*A*···N3	0.86	2.27	3.098 (7)	162
C1—H1*B*···O2	0.93	2.43	3.299 (8)	156
C9—H9*A*···O1	0.93	2.30	2.870 (8)	119
C17—H17*A*···O3	0.98	2.60	3.504 (8)	154
C19—H19*B*···N3^i^	0.97	2.58	3.538 (8)	170

Symmetry code: (i) −*x*+1, −*y*+1, −*z*+1.
